# UPLC fingerprinting combined with quantitative analysis of multicomponents by a single marker for quality evaluation of YiQing granules

**DOI:** 10.3389/fchem.2025.1632033

**Published:** 2025-07-28

**Authors:** YangHong Li, Bao Yu, Wei Zhao, Xiyu Pu, Xue Zhong, Xingbao Tao, Yunhong Wang, Weiguo Cao, Dan Zhang

**Affiliations:** ^1^ College of Traditional Chinese Medicine, Chongqing Medical University, Chongqing, China; ^2^ College of Chinese Materia Medica, Chongqing University of Chinese Medicine, Chongqing, China; ^3^ Chongqing Institute of Pharmaceutical Plant, Chongqing, China; ^4^ Yunnan University of Traditional Chinese Medicine, Kunming, Yunnan, China

**Keywords:** “YiQing” granule, quantitative analysis of multicomponents by a single marker (QAMS), UPLC, fingerprint, quantitative analysis, quality control

## Abstract

**Research background:**

YiQing granules (YQGs) are a patented drug commonly used in clinics in China. However, the quality control index of this preparation is relatively limited, which does not effectively ensure the quality of the product. Thus, a comprehensive quality evaluation of YQGs is lacking.

**Objective:**

The aim of this study was to establish a method for quantitative and qualitative analysis of YQGs based on ultra-high performance liquid chromatography-photodiode array detector (UPLC-PAD) fingerprint combined with quantitative analysis of multicomponents by a single marker (QAMS).

**Methods:**

We established and verified the comprehensive evaluation method that UPLC fingerprint combined with QAMS and stoichiometric method, to assess the overall quality of YQGs produced by different manufacturers. Berberine was selected as an internal reference, and the relative correction factors of coptisine, epiberberine, baicalin, berberine, palmatine, wogonoside, baicalein, wogonin, aloe-emodin, rhein, emodin, and chrysophanol were established.

**Study results:**

Results showed that the experimental time of the fingerprint was significantly reduced to approximately 0.5 h using the UPLC–PAD method. A total of 32 common peaks with similarity greater than 0.9 were identified. The accuracy of QAMS was compared with the external standard method, with no significant difference between the two methods.

**Conclusion:**

The UPLC fingerprint combined with QAMS method established in this study is feasible and reliable; it can be used for the comprehensive quality evaluation of YQGs and can provide a reference for the quality evaluation of other traditional Chinese medicine preparations.

## 1 Introduction

YiQing granules (YQGs) are a commonly used essential medicine that is listed in the Chinese Pharmacopoeia (2020 edition) and consists of *Coptis chinensis*, *rhubarb* and *Scutellaria baicalensis* (Commission, 2020). According to modern research, the main active components of *Coptis* are alkaloids, such as coptisine, epiberberine, palmatine, and berberine hydrochloride (Wang et al., 2019; Yang et al., 2021). The main active components of *Scutellaria baicalensis* are flavonoids, such as baicalin, wogonoside, baicalein, and wogonin (Wang et al., 2018; Li et al., 2015; Feng et al., 2010). The main active components of *rhubarb* are anthraquinones, such as aloe-emodin, rhein, emodin, and chrysophanol (Zhang et al., 2023; Zhang et al., 2021). Owing to YQGs’s heat-clearing, detoxifying, blood-cooling and blood-activating effects (Zheng et al., 2012; Liu et al., 2024), rhubarb has been clinically used for the treatment of sore throat, pharyngitis, amygdalitis, constipation, gingivitis, pharyngitis, and hemoptysis (Gong et al., 2017). Unlike the chemical drugs of higher purity, the traditional Chinese drug preparations are usually multi-component synergistic effects with compositional complexity. Therefore, more comprehensive quality control and evaluation methods are needed. Nowadays, fingerprint techniques have been widely used in the quality control of traditional Chinese medicine and its preparations.

Traditional Chinese medicine fingerprint is a quantifiable and comprehensive qualitative identification method based on the systematic study of the chemical components of traditional Chinese medicine (Wang et al., 2020; Gao et al., 2020). It can more comprehensively analyze the complex components in traditional Chinese medicine and thus control the overall quality of traditional Chinese medicine (Yang et al., 2023; Wu et al., 2021; Liu et al., 2022). This method has been approved by the World Health Organization, the United States Food and Drug Administration, the European Drug Administration (EMA), and China State Food and Drug Administration (Cuadros-Rodríguez et al., 2016; Zhang et al., 2024). However, there are some limitations in evaluating the quality of Chinese medicine or its preparations, and the characteristic common peak may not reflect the representative information of the quality of Chinese medicine and its preparations (Han et al., 2022; Li et al., 2021). In recent years, the stoichiometry analysis method has been widely used in chromatographic fingerprint analysis, such as similarity analysis (SA), hierarchical cluster analysis (HCA), principal component analysis (PCA), orthogonal partial least squares analysis (OPLS-DA) (Sun et al., 2019; Zhu et al., 2019; Si et al., 2020; Men et al., 2021), statistical analysis of fingerprint data, can effectively solve the problem that fingerprint cannot provide the representative composition of Chinese medicine quality control (Fan et al., 2022).

In Chinese Pharmacopoeia (2020 edition), the index component of YiQing particle quality control is only baicalin in *Scutellaria baicalensis*, and the corresponding content detection method has not been developed for *rhubarb* and *Coptis* (Chen et al., 2011). The complexity of Chinese medicine and its ingredients makes it difficult to evaluate the quality of a single component or index. Researchers have previously evaluated the quality of other YQGs with HPLC and UPLC–MS/MS. However, most studies have three key limitations: (1) reliance on single-indicator component analysis, which is difficult to comprehensively reflect the overall quality of traditional Chinese medicines; (2) dependence on control products, which leads to high testing costs; and (3) lack of multidimensional data integration capability, which prevents the full exploitation of the association patterns among quality markers. (Chen et al., 2011; Qu et al., 2007; Li et al., 2010; Zou et al., 2023). In most of the studies, multi-component quantitative analysis has been mainly conducted by the external standard method (ESM). However, some reference standards were unavailable and expensive. By contrast, quantitative analysis of multi-components by a single marker (QAMS) can realize the quantitative detection of multiple components using an inexpensive and easily accessible reference standard. QAMS (Zou et al., 2023; Wang et al., 2023; Xu et al., 2024; Zhang et al., 2022) for quantitative analysis of multiple components has been widely used in Chinese medicine (TCM) for quality control, this method not only solves the problem of unavailable or expensive reference standards, also greatly reduces the testing cost and time. At present, QAMS has been successfully applied to various Chinese medicinal materials and their preparations, such as the determination of active ingredients in eagle tea (Luo et al., 2023), tea products (Stekolshchikova et al., 2018), and Zuojin pill (Wang et al., 2023).

In this study, three key technologies were innovatively integrated: (a) UPLC was employed to achieve simultaneous separation of 12 components by optimizing the gradient elution procedure, which shortened the analysis time of traditional HPLC; (b) a QAMS multi-indicator quantitative method was established to achieve the alternative detection of difficult-to-access controls through the relative correction factor, which reduces the cost; and (c) chemometrics analysis was introduced (PCA, OPLS- DA, etc.), which revealed the synergistic effect pattern of the 3 pairs of components in the formulation. The comprehensive and scientific evaluation of the quality of YQGs not only provides a scientific basis and reference for the quality consistency control of YQGs for manufacturers and drug regulatory agencies but also provides a simple, fast, accurate, and reliable test method for the comprehensive evaluation of the quality of the drug.

## 2 Materials and methods

### 2.1 Instruments

Quantitative UPLC analysis was performed using a Waters e2695 ultrahigh-performance liquid chromatograph with a Waters 2998 PDA detector and Agilent 1290 Infinity II liquid chromatography. The chromatographic columns were Phenomenex Kinetex C18 column (2.1 mm × 50 mm, 1.7 μm), Waters ACQUITY UPLC BEH C18 column (2.1 mm × 50 mm, 1.7 μm), and Nuovasil SAB C18 column (2.1 mm × 50 mm, 1.8 μm).

An electronic balance was provided by Mettler Toledo. An SB-5200DT ultrasonic instrument was acquired from Ningbo Xinzhi Biotechnology Co., Ltd. An ultrapure water machine (Sartorius, Germany) was used in quantitative analysis.

### 2.2 Materials and reagents

Standards of emodin (batch number, MUST-23112011; purity, 98.09%), chrysophanic acid (batch number, MUST-23110720; purity, 99.46%), and rhein (batch number, MUST-23111012; purity, 99.1%) were originally purchased from Chengdu Manster Biotechnology Co., Ltd. (Sichuan, China). Standards of physcion (batch number, B20242; purity, ≥98%), rhabarberone (batch number, B20772; purity, ≥98%), baicalin (batch number, B20570; purity, ≥98%), and wogonoside (batch number, B20488; purity, ≥98%) were originally purchased from Yuanye Biotechnology Company, Ltd. (Shanghai, China). Standards of baicalein (batch number, 111595; purity, ≥98%), wogonin (batch number, 111595; purity, ≥98%), and berberine hydrochloride (batch number, 110713; purity, ≥98%) were originally purchased from the National Institutes for Food and Drug Control. Standards of epiberberine (batch number, 130413; purity, ≥98%), coptisine (batch number, 130809; purity, ≥98%), and palmatine chloride (batch number, 130614; purity, ≥98%) were originally purchased from Chengdu Pufield Biotechnology, Ltd. (Sichuan, China). Acetonitrile and methanol were purchased from Honeywell International (Shanghai, China) (HPLC grade). Phosphoric acid was purchased from Shanghai Aladdin Bio-Chem Technology Co., Ltd. (Shanghai, China) (HPLC grade). Triethylamine was purchased from Macklin Biochemical Co., Ltd. (Shanghai, China) (HPLC grade). Chemicals that are not mentioned here were of analytical grade.

Seventeen batches of YQGs (concentrated granules) were purchased from Taiji Group Co., Ltd. (23010002), ChongQing Peidu Pharmaceutical Co., Ltd. (230303), Shaanxi Dongtai Pharmaceutical Co., Ltd. (2L02 0201020), Guizhou Bailing Enterprise Group Zhengxin Co., Ltd. (20230209), Shijiazhuang No. 4 Pharmaceutical Co., Ltd. (YQ23060901), Sichuan Diite Pharmaceutical Co., Ltd. (320502), Inner Mongolia Tianqi China–Mongolia Pharmaceutical Co., Ltd. (22303010), Hubei Newland Pharmaceutical Co., Ltd. (20240301), Shanxi Huayuan Pharmaceutical Biotechnology Co., Ltd. (230105), Shaanxi Pharmaceutical Holding Group Co., Ltd. (230201), Jilin Jinghui Pharmaceutical Co., Ltd. (230303 2025), Jilin Aodong Group Jinhaifa Pharmaceutical Co., Ltd. (230704), Jilin Yimintang Pharmaceutical Co., Ltd. (240101), Heilongjiang Linhai Xueyuan Pharmaceutical Co., Ltd. (20230101), Tonghua Jinkai Pharmaceutical Co., Ltd. (221212), Zhengzhou Furuitang Pharmaceutical Co., Ltd. (231207), and ZhongZhi Pharmaceutical Group (20240506).

### 2.3 Preparation of mixed chemical standard solution and sample solution

#### 2.3.1 Preparation of mixed standard solutions

The appropriate amount of reference substances of coptisine, epiberberine, baicalin, berberine hydrochloride, palmatine, wogonoside, baicalein, wogonin, aloe-emodin, rhein, emodin, and chrysophanol were accurately weighed and dissolved in methanol accordingly. Then, the obtained mixture of standards was diluted to the appropriate concentration ranges. The calibration curves were plotted with the concentration on the X-axis and the peak area on the Y-axis. All solutions were stored at −20°C.

#### 2.3.2 Sample solution preparation

YQGs were crushed using a mortar. Then, 1.0000 g of fine powder of YQGs was accurately weighed and placed in a 50 mL measuring flask, and then, approximately 30 mL of methanol was added. The mixture was sonicated for 30 min, cooled, made up for the weight loss with methanol, and filtered using a 0.22 μm microporous membrane. The subsequent filtrate was taken as the sample solution. All solutions were stored at −20°C.

### 2.4 Chromatographic conditions

**FIGURE 1:**
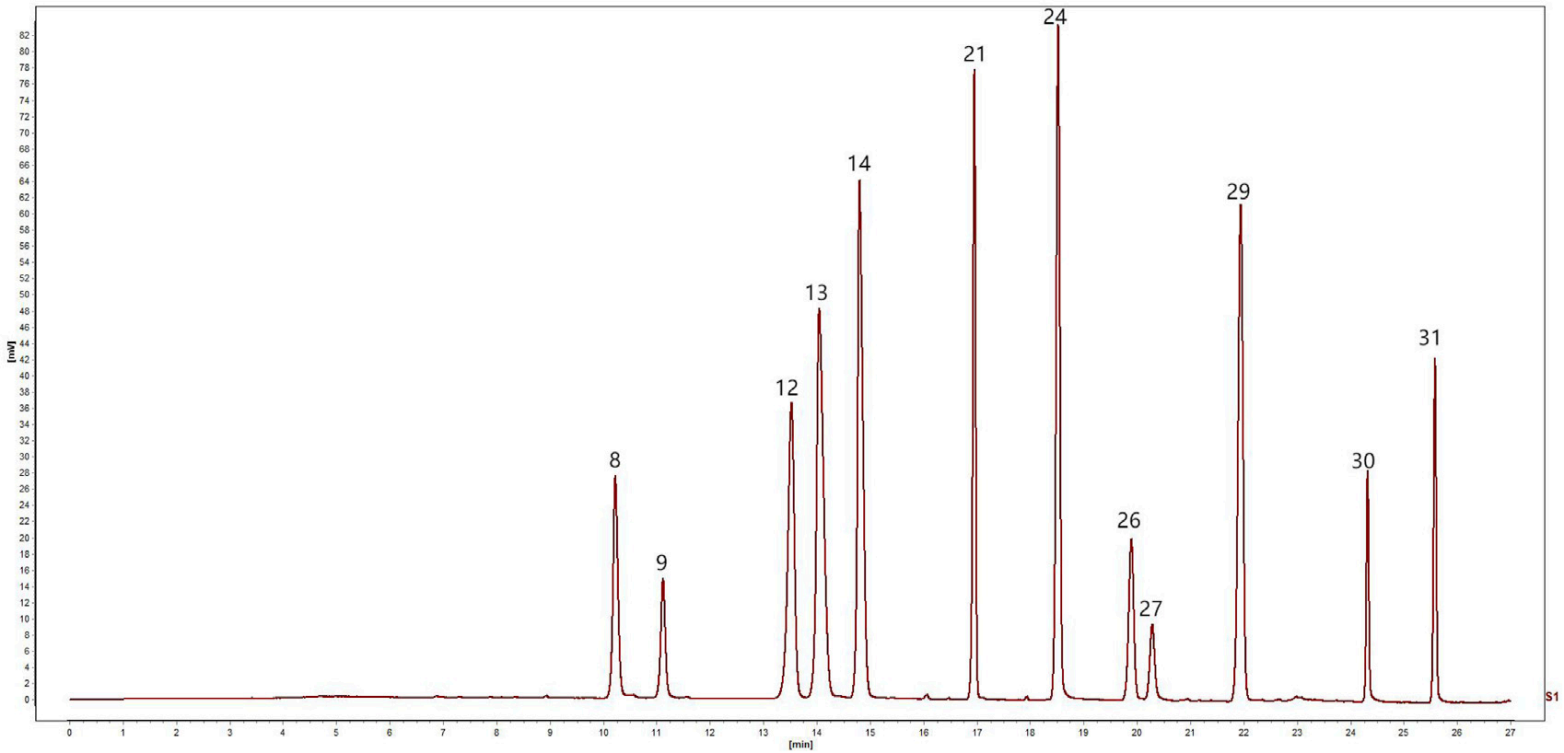
UPLC chromatogram of mixed reference solution: 8, Coptisine, 9, Epiberberine, 12, Baicalin, 13, Berberine, 14, Palmatine, 21, Wogonoside, 24, Baicalein, 26, Aloe-emodin, 27, Rhein, 29, Wogonin, 30, emodin, 31, Chrysophanol.

The mobile phase was acetonitrile (B)–0.2% phosphoric acid (triethylamine tone PH was 3) (A) with gradient elution (0–5 min, 5% B→10% B; 5–11 min, 10%→15% B; 11–13 min, 15% B; 13–17 min, 15% B→25% B; 17–19 min, 25% B→27% B; 19–21 min, 27% B→30% B; 21–26 min, 30% B→60% B; 26–27 min, 60% B→90% B). The flow rate was 0.4 mL/min. Here, 254, 276, and 345 nm were set as detection wavelengths, the column temperature was 35°C, and the injection volume was 1 μL. The chromatograms of the mixed standard solution under the abovementioned chromatographic conditions are shown in Figure 1.

### 2.5 UPLC method and validation

This study verified the regression equation, correlation coefficient, linear range, limit of detection (LOD), limit of quantification (LOQ), precision, recovery, and stability to assess whether the method meets the requirements of the Chinese Pharmacopoeia.

Mixed standard solutions of different concentrations were injected into the UPLC system, recording the profiles and drawing the calibration curve using the peak area (Y) versus the concentration (X) of the compound. The LOD and LOQ were measured based on the signal-to-noise ratio of 3 and 10, respectively.

Precision was tested using six consecutive injections of the mixed standard solution. S10 was randomly selected as the trial sample. Stability was determined by sequential injection of the sample solution after 0, 2, 4, 8, 12, and 24 h. Reproducibility was determined by injecting six S10 sample solutions extracted in parallel. Sample recovery was measured by adding equal concentrations of control materials to samples of known concentrations (n = 6).

### 2.6 Quantitative analysis of multicomponents by a single marker

The commonly used methods for calculating relative correction factor (RCF) include multipoint correction (Luo et al., 2023; Xiong et al., 2014; Huang et al., 2018), slope correction (Zeng et al., 2024; Peng et al., 2018), and a linear method (Wang et al., 2015). In this study, the correction factor was calculated using the slope method. The RCF between the internal reference object and each component to be measured was calculated using Formula 1. The obtained RCF was taken as a constant, and the amount of the components to be measured was calculated using Formulas 2, 3.

$$f_{\text{ki}}=\frac{f_{k}}{f_{i}},$$ (1)

$$C_{i}=f_{\text{ki}}\times C_{K}\times \frac{A_{i}}{A_{k}},$$ (2)

$$W\left(\frac{\text{mg}}{g}\right)=\frac{C_{i}\times V}{m}\times 1000,$$ (3)

where f_i_ is the slope of the standard curve of the internal reference control, f_k_ is the slope of the standard curve of the control of a component to be tested, A_i_ is the peak area of the component in the test article, C_i_ is the concentration of the tested component in the test article, A_k_ is the peak area of the reference in the test article, and C_k_ is the concentration of the reference in the test article. W is the content of the components in YQGs (mg/g), m is the weight of YQGs, and V is the volume of the YQG solution.

Quality control of YQGs was performed using the QAMS method, which calculates RCF based on the ratio of each standard to the detector within a certain range. RCF values were calculated with different injection concentrations and the stability of RCF in the study under different conditions.

Quality control of YQGs was performed using the QAMS method, which was according to the proportional value of each standard to the detector within a certain range to calculate RCFs. In this study, the chromatographic columns (Phenomenex Kinetex C18 column, Waters ACQUITY UPLC BEH C18 column, and Nuovasil SAB C18 column), column temperature (33°C, 35°C, and 37°C), and flow rates (0.36, 0.4, and 0.44 mL/min) of the mobile phase were investigated. The internal reference material should be economical, stable, less toxic, and effective, with good separation under appropriate chromatographic conditions (Yang et al., 2023; Li et al., 2010).

### 2.7 Comparison of QAMS measurement results with the external standard method (ESM)

Standard method deviation (SMD) was calculated using QAMS and the ESM to evaluate the accuracy of the calculation results of the QAMS method.

$$\text{SMD}\left(\%\right)=\frac{C_{\text{ESM}}-C_{\text{QAMS}}}{C_{\text{ESM}}}\times 100\%,$$ (4)

where C_ESM_ and C_QAMS_ represent the contents of the analytes assayed by the ESM and QAMS method, respectively.

### 2.8 Establishment of UPLC fingerprint and scientific analysis of data

The 17 batches of YQGs were prepared according to the preparation method of the sample solution. The samples were determined, and the chromatograms were recorded. After the unified integration of all chromatograms, the obtained chromatograms were imported into the “Similarity Evaluation System for Chromatographic Fingerprint of Traditional Chinese Medicine (2012 Edition)” software in AIA format to establish the UPLC fingerprint. The similarity of the fingerprints of the 17 batches of YQGs was also calculated using the abovementioned software.

Excel 2019 was used for statistical analysis of the obtained data. With the content of 12 kinds of compounds as variables, cluster analysis was performed on the 17 batches of the samples. SPSS (27.0.1) and SIMAC (14.0) were used for HCA and PCA, respectively.

## 3 Results and discussion

### 3.1 Linear regression analysis

Linear regression analysis was conducted with the concentration of the control article as the abscissa (X) and the peak area as the ordinate (Y). As shown in the results in Figure 1, all the peaks were separated well within 27 min. Within the test range, the correlation coefficients of the calibration curves for the 12 components ranged between 0.9997 and 1.0000, showing a good linear relationship (Table 1).

**TABLE 1:** Calibration curve, linear range, LOD and LOQ for standards.

Component	Linear equation	R^2^	Linear range (μg/mL)	LOD (μg/mL)	LOQ (μg/mL)
Epiberberine	y = 8486.8x - 3429	0.9998	0.403–25.811	0.499	1.512
Coptisine	y = 7701.8x - 856.94	0.9999	0.285–18.22	0.151	0.457
Palmatine	y = 12038x - 5448.6	1.0000	1.186–75.871	0.747	2.262
Berberine	y = 12992x - 1571	0.9999	0.400–25.621	0.373	1.131
Baicalin	y = 11669x + 21286	0.9999	4.051–259.243	4.050	12.272
Wogonoside	y = 14441x + 5919.5	0.9998	0.793–50.718	0.949	2.877
Baicalein	y = 21699x - 2446.9	0.9997	0.261–16.670	0.389	1.178
Wogonin	y = 21970x + 510.54	0.9999	0.0870–5.570	0.080	0.244
Aloe-emodin	y = 21380x + 67.73	0.9998	0.052–3.347	0.056	0.171
Rhein	y = 6932.4x - 871.32	0.9998	0.268–17.146	0.346	1.050
Emodin	y = 12044x + 25.649	1.0000	0.031–1.994	0.089	0.269
Chrysophanol	y = 17829x + 341.77	0.9998	0.061–3.895	0.070	0.212

### 3.2 Methodology verification

According to the guidelines of the Chinese Pharmacopoeia approach, system precision, repeatability, stability, and recovery were determined to verify the feasibility of the UPLC method. The retention time and peak area of each common peak were calculated using the precision, repeatability, stability, and recovery tests. Results showed that the relative standard deviation (RSD) of the sample recovery ranged from 96.86% to 101.05% and repeatability was between 1.21% and 2.35%, the RSD of precision was less than 1.47%, and stability was less than 0.78% (Table 2), indicating that the established method was sufficient for the qualitative and quantitative analysis of the 12 compounds selected.

**TABLE 2:** The methodological verification of UPLC analytical methods.

Component	Precision RSD (%)	Stability RSD (%)	Repeatability RSD (%)	Accuracy	
				Recovery rate (%)	RSD (%)
Epiberberine	1.08	0.36	1.32	100.05	3.50
Coptisine	1.20	0.41	1.25	101.05	3.89
Palmatine	1.15	0.23	1.29	98.79	2.16
Berberine	1.22	0.74	1.45	101.05	3.28
Baicalin	1.19	0.28	1.21	98.41	1.90
Wogonoside	1.19	0.28	1.24	98.00	2.78
Baicalein	1.36	0.25	1.51	96.86	1.96
Wogonin	1.47	0.71	1.63	100.56	2.72
Aloe-emodin	1.32	0.46	1.22	98.31	2.82
Rhein	1.31	0.47	1.27	98.03	3.65
Emodin	0.88	0.78	1.46	100.92	1.96
Chrysophanol	1.14	0.41	2.35	100.06	2.90

### 3.3 UPLC fingerprint and SA

**FIGURE 2:**
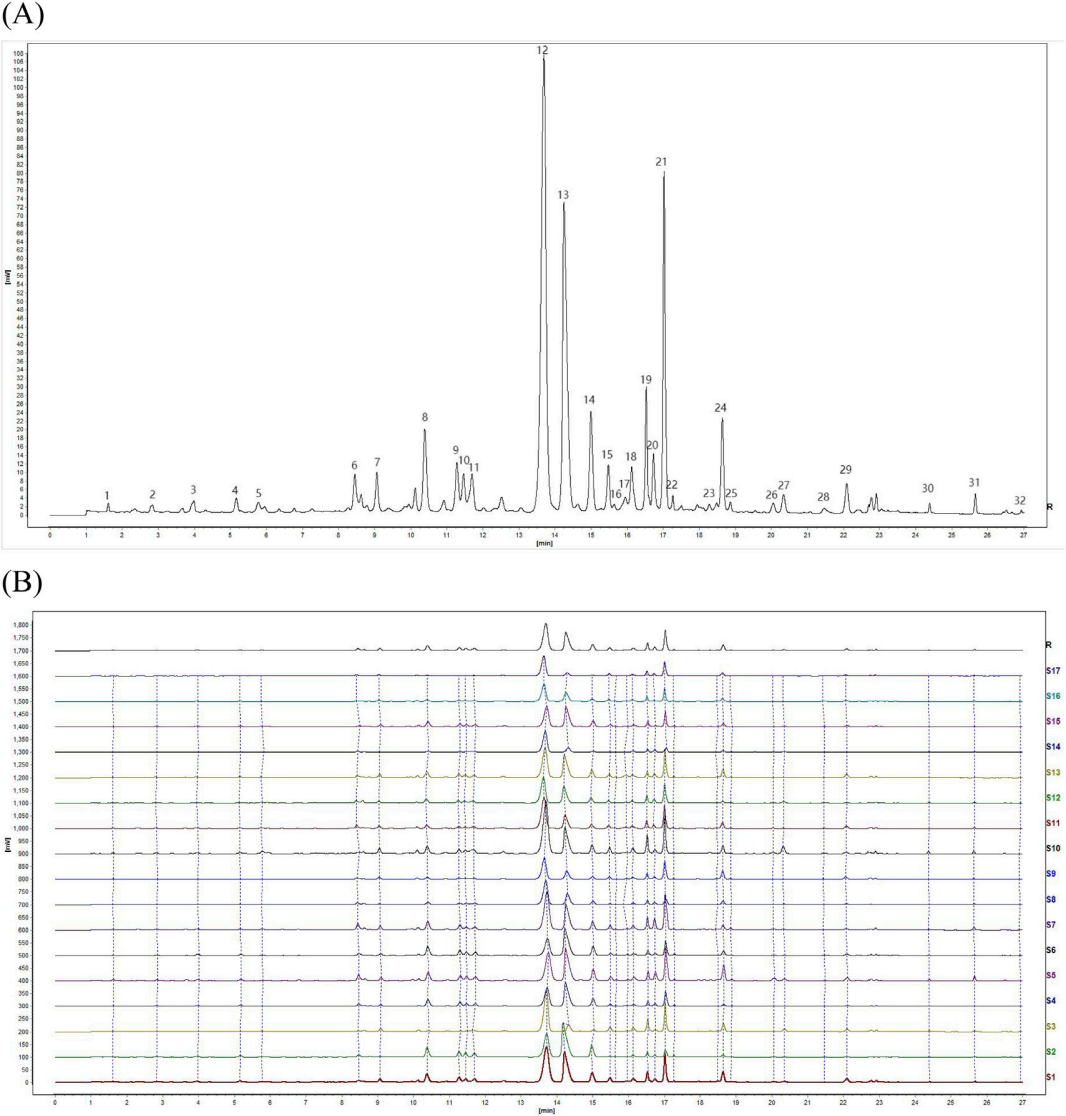
Fingerprints of 17 batches of YiQing granules (YQGs) at 254 nm. **(A)** Identification of chemical common peaks; **(B)** UPLC fingerprints and reference fingerprint (R).

The UPLC chromatograms of the 17 batches of YQGs were generated using the “Similarity Evaluation System for Chromatographic Fingerprint of Traditional Chinese Medicine” (version 2012A). With the S8 sample as the reference fingerprint, the median method was adopted, with a time window width of 0.1, and the control fingerprint was generated after multipoint correction and automatic matching (Figure 2A). Then, similarity was calculated. The results showed that the fingerprint of the YQG samples from S1–S17 were similar (Figure 2B). The RSD of the relative retention time (RRT) of each common peak was lower than 0.43%, and the RSD of the relative peak area of each common peak was lower than 5.85%. The SA value between samples was >0.9 (Table 3), indicating that the chemical composition of YQGs is similar among different manufacturers, and the overall quality of the samples is not different. It also indicates that the difference between samples of different manufacturers cannot be accurately judged by similarity, and further stoichiometry analysis is needed to reflect the internal quality of YQGs more objectively. On the basis of the analytical chromatograms, a standard fingerprint was established, and 32 stable and reproducible common peaks were identified. Based on their UPLC retention times by comparing with standard compounds and combined with spectroscopic analysis, a total of 12 compounds were identified, including peaks 8, 9, 12, 13, 14, 21, 24, 26, 27, 29, 30, and 31, which were identified as coptisine, epiberberine, baicalin, berberine, palmatine, wogonoside, baicalein, aloe-emodin, rhein, wogonin, emodin, and chrysophanol, respectively (1).

**TABLE 3:** Similarity results of UPLC fingerprints of YiQing Granules.

Batch	Similarity	Batch	Similarity
S1	0.997	S10	0.985
S2	0.903	S11	0.983
S3	0.921	S12	0.997
S4	0.947	S13	0.997
S5	0.972	S14	0.917
S6	0.901	S15	0.983
S7	0.990	S16	0.993
S8	0.973	S17	0.922
S9	0.966		

### 3.4 Hierarchical clustering analysis

**FIGURE 3:**
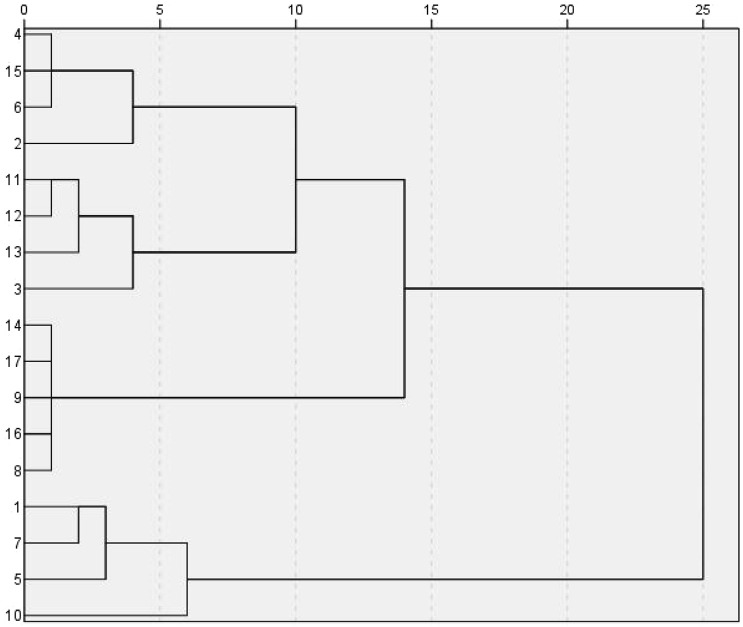
Dendrograms of HCA for the 17 tested samples of YQGs.

Using SPSS 27.0 software, the common peak area values of 32 index components in the 17 batches of samples were used as the input index. Cluster analysis used the Wald method and Euclidean distance as the metrics, and the vertical orientation lineage map was the output. The short distance between two samples in the HCA dendrogram indicates high similarity, and the samples clustered into the same group have the greatest similarity. According to the correlation coefficients among 17 batches of samples, Seventeen YQGs samples from different pharmaceutical companies, were divided into two categories, S1, S5, S7, S10 clustered into one category, and the rest of the pharmaceutical companies clustered into another category (Figure 3). The results show that YQGs produced by different manufacturers were different in composition and content. YQGs of adjacent batches may be more similar in their source and processing methods and therefore more likely to be grouped together. The production conditions of different manufacturers are not identical. Therefore, it is reasonable to have some quality differences between different batches of samples. Moreover, the YQGs produced by each manufacturer may be affected by the source of prescription materials, processing methods, production process, and transportation and storage conditions, leading to quality differences.

### 3.5 Principal component analysis

**FIGURE 4:**
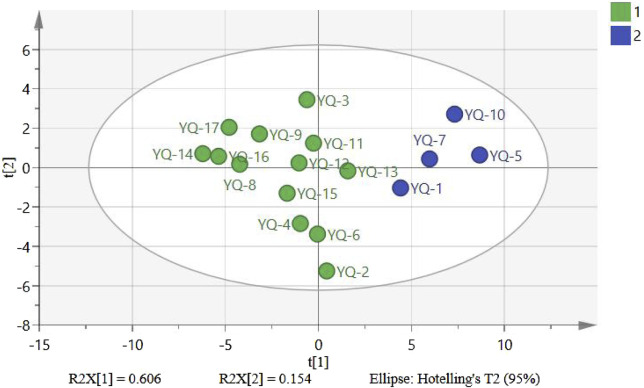
Technical analysis of fingerprint in 17 batches of YQGs.

PCA is a stoichiometry method used for feature extraction and dimensionality reduction. It is widely used to reduce a large number of regional variables to an appropriate number of principal components that are orthogonal and account for the maximum percentage of variance in the data (Trawiński and Skibiński, 2019; Gan et al., 2019). For reasonable evaluation and comprehensive analysis of the chemical composition from different companies, the data of 17 tested samples were imported into SIMCA14.0 software, the four principal components (PC1, PC2, PC3, and PC4) contained the most information of all variables, and the cumulative contribution of the four components was 90.4%, which can represent most of the information of the 32 common peaks in the fingerprint. PC1 and PC2 had eigenvalues of 10.3 and 2.62, representing 60.6% and 15.4% of the total variance, respectively. As shown in Figure 4, 17 batches of samples can be divided into two groups, S1, S5, S7, S10, and the remaining batch one, the results of the scatter plots were consistent with that of HCA. The scatter plot clearly shows the quality differences of YQGs from different manufacturers. This may be caused by the uncertainty of the harvest season, harvest origin, processing method, and the production environment of the medicinal materials selected by different manufacturers. It is suggested that drug manufacturers should pay attention to the source of original herbal medicines, improve the internal control quality standards of preparations, and make the quality of preparations more stable and effective.

### 3.6 Orthogonal partial least squares discrimination analysis (OPLS-DA)

**FIGURE 5:**
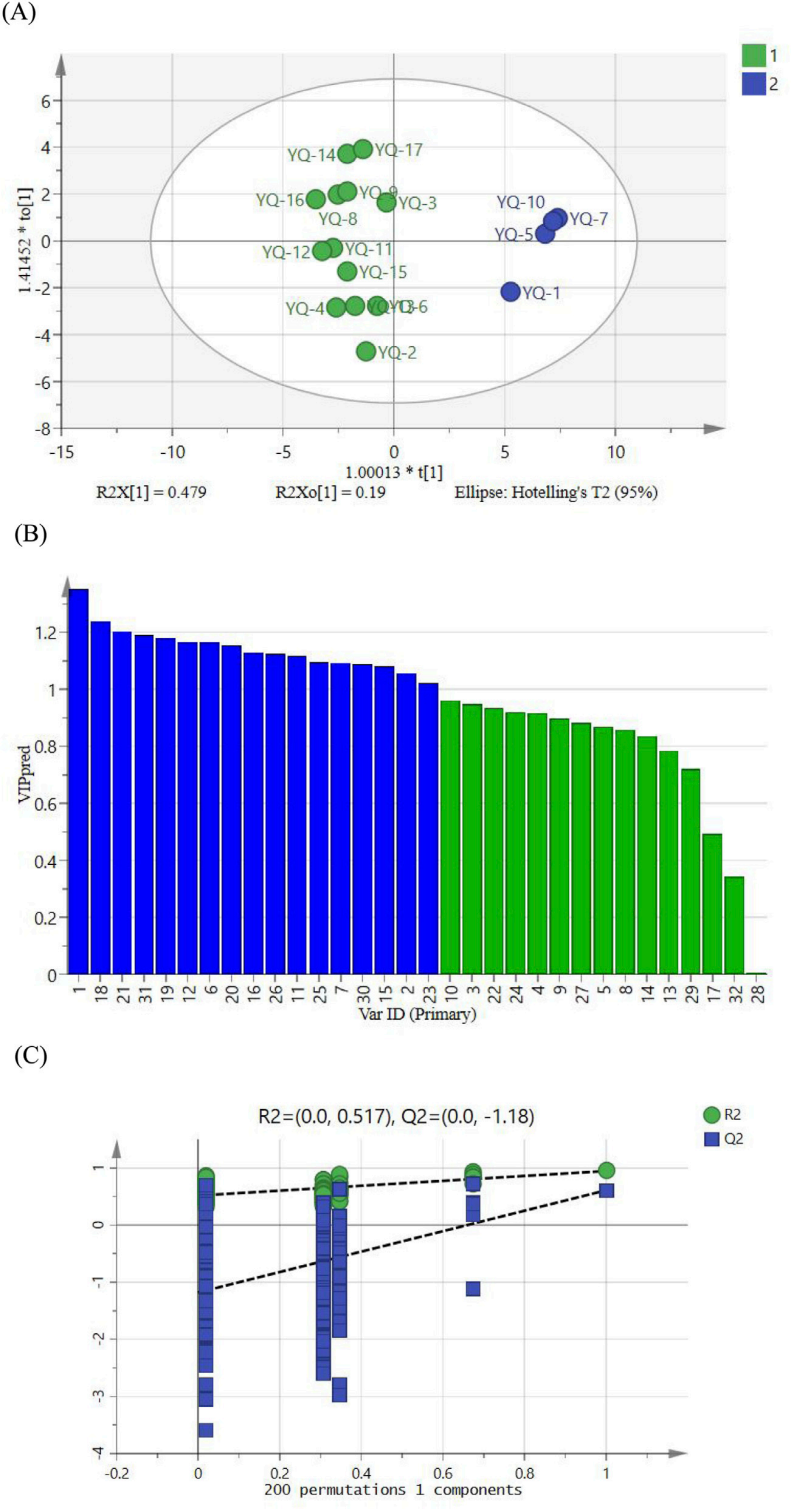
**(A)** Plot of OPLS-DA scores for YQGs of different manufacturers. **(B)** VIP values for 32 shared peaks. **(C)** Model cross-validation plot.

To further explore the differences between 17 batches of YQGs, based on the PCA results, orthogonal partial least squares discriminant analysis (OPLS-DA) was used based on the VIP value of the model. The OPLS-DA model had a fit index of 0.842 for the independent variables (R^2^x), a fit index of 0.946 for the dependent variables (R^2^y), and a predictive index (Q^2^) of 0.609. Both R^2^ and Q^2^ surpassing 0.5, validated the model’s reliability. The OPLS-DA score plot was shown in Figure 5A. The 17 batches of samples can be well divided into two groups, which are consistent with the results of HCA and PCA. After the 200 permutation tests depicted in Figure 5B, higher R^2^ and Q^2^ values on the right side and the Q^2^ regression line intercepting below 0 (−1.18) affirmed the model’s validation without overfitting, ensuring its utility in identifying differential markers. It is generally believed that variables with VIP >1 play a key role in classification. As can be seen from the 5 (C), 17 differential markers were identified, including peaks 1, 18, 21, 31, 19, 12, 20, 6, 16, 26, 11, 25, 7, 30, 15, 2, and 23. Among them, peaks 21, 31, 12, 26, and 30 were identified as wogonoside, chrysophanol, baicalin, aloe-emodin, and emodin, which can be used as markers affecting the quality of YQGs. These components are the main characteristic components that cause the differences between different manufacturers of YQGs and are crucial for distinguishing samples and classification.

### 3.7 Results of RCF determination

After comparison, among the components to be tested, berberine had a high content, stable properties, the best chromatographic peak shape, and the lowest standard cost and was nontoxic and easy to obtain. Thus, berberine was selected as the internal standard. The standard mixture was diluted step by step to obtain a gradient series of mixed standard solutions numbered 1–7. Berberine was selected as the reference compound, and the positive slope ratio of the standard curve slope of berberine to the components to be tested was used as the RCF value. According to the ratio, the RCFs for coptisine, epiberberine, palmatine, baicalin, wogonoside, baicalein, wogonin, aloe-emodin, rhein, emodin, and chrysophanol were 1.418, 1.563, 0.927, 1.032, 0.834, 0.555, 0.548, 0.563, 1.736, 1.000, and 0.675, respectively.

### 3.8 Durability of RCF

Instrument, chromatographic columns, temperature, and flow velocity are usually the most essential factors affecting the RCF (Zhu et al., 2017). The RCFs of the other 11 components were calculated for different instruments, columns, temperature, and flow rates, using berberine as the internal reference. As shown in Table 4, the RSDs of all 11 compounds relative to the RCF of berberine were less than 5%, with good repetition and good robustness.

**TABLE 4:** Results of the calculations of the RCF of 12 components.

Condition		f_Coptisine_	f_Epiberberine_	f_Palmatine_	f_Baicalin_	f_Wogonoside_	f_Baicalein_	f_Wogonin_	f_Aloe-emodin_	f_Rhein_	f_Emodin_	f_Chrysophanol_
flow rate (mL/min)	0.39	1.412	1.554	0.926	1.030	0.833	0.553	0.546	0.551	1.729	0.997	0.666
	0.4	1.418	1.563	0.927	1.032	0.834	0.555	0.548	0.563	1.736	1.000	0.675
	0.41	1.429	1.556	0.926	1.030	0.831	0.550	0.547	0.560	1.742	1.001	0.664
Temperature (°C)	33	1.419	1.550	0.922	1.030	0.831	0.550	0.544	0.545	1.778	0.986	0.667
	35	1.418	1.563	0.927	1.032	0.834	0.555	0.548	0.563	1.736	1.000	0.675
	37	1.418	1.567	0.930	1.033	0.834	0.549	0.550	0.561	1.738	0.996	0.665
Waters	Nuovasil	1.411	1.563	0.925	1.073	0.844	0.568	0.544	0.568	1.721	0.985	0.685
	Kinetex	1.418	1.563	0.927	1.032	0.834	0.555	0.548	0.563	1.736	1.000	0.675
	Waters	1.416	1.560	0.923	1.030	0.838	0.563	0.541	0.554	1.731	0.994	0.681
Agilent	Nuovasil	1.418	1.569	0.927	1.022	0.830	0.556	0.545	0.565	1.729	0.996	0.669
	Kinetex	1.416	1.567	0.925	1.023	0.820	0.549	0.547	0.561	1.735	0.992	0.666
	Waters	1.418	1.562	0.923	1.031	0.829	0.550	0.546	0.562	1.733	0.997	0.661
Mean		1.418	1.561	0.926	1.033	0.833	0.554	0.546	0.560	1.737	0.995	0.671
RSD%		0.310	0.360	0.241	1.260	0.678	1.061	0.440	1.159	0.805	0.534	1.099

### 3.9 Location of the analytes’ chromatographic peaks

The premise of realizing one test and multiple evaluation is to use effective means to accurately locate the corresponding chromatographic peaks of the measured components. At present, the RRT or RTT difference is commonly used to locate chromatographic peaks (Mai et al., 2020). The range of the RTT difference between the target analyte and the reference by the method is large, and the data fluctuate greatly (Luan et al., 2018). Therefore, the RRT was used for peak positioning in this study. Different instruments and columns were used to determine the RRT of coptisine, epiberberine, palmatine, berberine, baicalin, wogonoside, baicalein, wogonin, aloe-emodin, rhein, emodin, and chrysophanol. RRT, mean RRT, and RSD were calculated separately for each of the above components. The results in Table 5 indicate less fluctuating RRT for measurements across different instruments and columns. The RSD values of all 12 fractions were less-than-or-equal-to 5.0%. Thus, the relative retention value can be used for locating the chromatographic peaks of the test components.

**TABLE 5:** Relative retention times (RRTs) by different columns and different instruments.

Components	Waters			Agilent			Mean	RSD%
	Nuovasil	Kinetex	Waters	Nuovasil	Kinetex	Waters		
Epiberberine	0.732	0.727	0.780	0.726	0.725	0.765	0.743	3.217
Coptisine	0.784	0.789	0.834	0.777	0.786	0.816	0.798	2.767
Palmatine	1.038	1.052	1.016	1.041	1.059	1.015	1.037	1.742
Baicalin	0.971	0.953	0.970	0.958	0.952	0.963	0.961	0.861
Wogonoside	1.182	1.195	1.116	1.196	1.232	1.117	1.173	3.989
Baicalein	1.291	1.307	1.245	1.315	1.357	1.253	1.295	3.218
Wogonin	1.508	1.549	1.433	1.544	1.615	1.452	1.517	4.443
Aloe-emodin	1.394	1.405	1.363	1.424	1.461	1.380	1.404	2.471
Rhein	1.447	1.427	1.391	1.464	1.482	1.395	1.434	2.562
Emodin	1.681	1.714	1.557	1.722	1.760	1.580	1.669	4.926
Chrysophanol	1.765	1.803	1.647	1.814	1.875	1.674	1.763	4.953

### 3.10 Comparison of content determination results between QAMS and ESM

As shown in Supplementary Table S1, the QAMS calculation of 12 compounds and the SMD results were less than or equal to 5.002%, with no significant difference between the two methods. SMD is calculated using Formula 4. Therefore, the QAMS method that we established is accurate, can be used to determine the content of 12 compounds in single particles simultaneously, and can greatly reduce the detection cost and improve the detection efficiency.

## 4 Conclusion

In this study, 0.1% aqueous formic acid solution–methanol, 0.1% aqueous phosphate solution–methanol, 0.2% aqueous phosphate solution–acetonitrile, and 0.2% aqueous phosphate solution–methanol were investigated as the mobile phases. However, the alkaloid components were towed, and the separation effect was poor. Therefore, triethylamine was used to adjust the mobile phase pH to improve the peak shape and separation. As a result, the map quality of the 0.2% aqueous phosphate solution (triethylamine adjusted pH to 3)–acetonitrile system was the best, and the RTT of the peaks was more moderate. Therefore, it is the best chromatographic condition.

The QAMS method established in this study could determine the contents of four alkaloids (coptisine, epiberberine, berberine, and palmatine), four flavonoids (baicalin, wogonoside, baicalein, and wogonin), and four anthraquinones (aloe-emodin, rhein, emodin, and chrysophanol) under the same chromatographic conditions. Using berberine as the internal standard, the contents of the active ingredients in 12 of 17 batch YQGs were determined simultaneously using RCF, which verified that this method was feasible. Results showed no significant difference between the established QAMS method and ESM. QAMS utilizes a single internal reference standard to accurately quantify multiple target compounds simultaneously. First of all, compared with the traditional external standard method (each compound requires its own standard), QAMS significantly reduces the cost, especially for expensive or scarce standards. Secondly, QAMS significantly improves analytical efficiency (simultaneous detection of 12 across-class active ingredients), simplifies the experimental process, and shortens the analytical cycle time. Finally, the method breaks through the traditional limitation of “one standard, one test”, effectively overcomes the problem of scarcity of standards, breaks through the dependence on the accessibility of pure products for all targets, and greatly expands the feasibility and practicability of multi-component quantitative analysis in complex systems (e.g., traditional Chinese medicine, natural products).

In addition, we established the UPLC fingerprint of YQGs (combined with SA, HCA, PCA, and OPLS-DA) to identify and evaluate their quality. In this study, according to a series of stoichiometric methods based on 32 common peaks, YQGs from different manufacturers were divided into two groups, S1, S5, S7, S10, and the remaining batch one. Based on the OPLS-DA analysis, five mass markers (wogonoside, baicalin, aloe-emodin, emodin, and chrysophanol) were identified in YQGs. The results show that the consistency of the YQGs of different manufacturers is still different, we believe that the possible reasons for the inconsistency of the quality of YQGs from different pharmaceutical companies are differences in the sources of herbal medicines, such as the appropriateness of the picking time of original herbs, or the use of the processing method of original herbs. The particle preparation process, storage, and transportation conditions are also one of the essential factors affecting the quality of YQGs.

In this study, the multi-technology coupling strategy not only solves the limitations of the existing methods, but more importantly, establishes a new paradigm of quality control of ‘ingredient detection-quality assessment’, which provides an innovative methodological reference for quality standardization studies of traditional Chinese medicine. This new method will provide a strong basis for the quality evaluation of YQGs and can achieve the purpose of comprehensive quality evaluation, which is more comprehensive than the evaluation of a single index. It can help inspection and testing personnel effectively improve the efficiency and accuracy of daily inspection work, provide technical support for the quality evaluation and control of different batches of different manufacturers, can be used by regulators for batch release inspection, reducing reliance on single ingredient testing, and provide an essential reference for the rapid evaluation of other related Chinese patent medicines.

## Data Availability

The original contributions presented in the study are included in the article/Supplementary Material, further inquiries can be directed to the corresponding authors.

## References

[B1] Chen J. Yang Y. Shi Y. P. (2011). Simultaneous quantification of twelve active components in yiqing granule by ultra‐performance liquid chromatography: application to quality control study. Biomed. Chromatogr. 25 (9), 1045–1053. 10.1002/bmc.1569 21154891

[B2] Commission S. P. (2020). Pharmacopoeia of the people’s republic of. China. Beijing: China medical science press.

[B3] Cuadros-Rodríguez L. Ruiz-Samblás C. Valverde-Som L. Pérez-Castaño E. González-Casado A. (2016). Chromatographic fingerprinting: an innovative approach for food'identitation'and food authentication–A tutorial. Anal. Chim. Acta. 909, 9–23. 10.1016/j.aca.2015.12.042 26851080

[B4] Fan X. Hong T. Yang Q. Wang D. Peng J. Xiao W. (2022). Quality assessment of fried licorice based on fingerprints and chemometrics. Food. Chem. 378, 132121. 10.1016/j.foodchem.2022.132121 35032797

[B5] Feng J. Xu W. Tao X. Wei H. Cai F. Jiang B. (2010). Simultaneous determination of baicalin, baicalein, wogonin, berberine, palmatine and jatrorrhizine in rat plasma by liquid chromatography–tandem mass spectrometry and application in pharmacokinetic studies after oral administration of traditional Chinese medicinal preparations containing scutellaria–coptis herb couple. J. Pharm. Biomed. Anal. 53 (3), 591–598. 10.1016/j.jpba.2010.04.002 20430560

[B6] Gan Y. Xiao Y. Wang S. Guo H. Liu M. Wang Z. (2019). Protein-based fingerprint analysis for the identification of ranae oviductus using RP-HPLC. Molecules 24 (9), 1687. 10.3390/molecules24091687 31052194 PMC6539769

[B7] Gao L. Wang F. Meng M. (2020). Chromatographic fingerprinting and quantitative analysis for the quality evaluation of xinfeng capsule. Acta. Chromatogr. 33 (1), 37–43. 10.1556/1326.2020.00743

[B8] Gong D. Hong Y. Sun G. Zhang J. (2017). Novel strategy for quality consistency evaluation of Chinese medicine “YIQING” tablet that combines the simultaneous quantification and screening of ten bioactive constituents. J. Sep. Sci. 40 (15), 3064–3073. 10.1002/jssc.201700291 28590083

[B9] Han J. Xu K. Yan Q. Sui W. Zhang H. Wang S. (2022). Qualitative and quantitative evaluation of Flos puerariae by using chemical fingerprint in combination with chemometrics method. J. Pharm. Anal. 12 (3), 489–499. 10.1016/j.jpha.2021.09.003 35811625 PMC9257449

[B10] Huang J. Yin L. Dong L. Quan H. Chen R. Hua S. (2018). Quality evaluation for Radix astragali based on fingerprint, indicative components selection and QAMS. Biomed. Chromatogr. 32 (11), e4343. 10.1002/bmc.4343 30003570

[B11] Li C. Tian Y. Zhao C. Li S. Wang T. Qiao B. (2021). Application of fingerprint combined with quantitative analysis and multivariate chemometric methods in quality evaluation of dandelion (*Taraxacum mongolicum*). Sci. 8 (10), 210614. 10.1098/rsos.210614 PMC8548788 34729206

[B12] Li Y. Wu T. Zhu J. Wan L. Yu Q. Li X. (2010). Combinative method using HPLC fingerprint and quantitative analyses for quality consistency evaluation of an herbal medicinal preparation produced by different manufacturers. J. Pharm. Biomed. Anal. 52 (4), 597–602. 10.1016/j.jpba.2010.01.018 20138726

[B13] Li Y. X. Gong X. H. Li Y. Zhang R. Q. Yuan A. Zhao M. J. (2015). The influence of Aconitum carmichaelii debx. On the pharmacokinetic characteristics of main components in Rheum palmatum L. Phytother. Res. 29 (8), 1259–1264. 10.1002/ptr.5369 25963314

[B14] Liu X. Yang T. Chen L. Lan L. Sun G. Guo P. (2024). A strategy takes “Yiqing” tablets as an example to carry out simpler multi-component quantification and use fingerprint technology for comprehensive quality consistency evaluation. J. Pharm. Biomed. Anal. 238, 115809. 10.1016/j.jpba.2023.115809 37944458

[B15] Liu Z. Qu J. Ke F. Zhang H. Zhang Y. Zhang Q. (2022). Material basis elucidation and quantification of dandelion through spectrum–effect relationship study between UHPLC fingerprint and antioxidant activity via multivariate statistical analysis. Molecules 27 (9), 2632. 10.3390/molecules27092632 35565983 PMC9101216

[B16] Luan L. Shen X. Liu X. Wu Y. Tan M. (2018). Qualitative analysis of psoraleae fructus by HPLC‐DAD/TOF‐MS fingerprint and quantitative analysis of multiple components by single marker. Biomed. Chromatogr. 32 (2), e4059. 10.1002/bmc.4059 28777876

[B17] Luo J. Cao W. G. Yu B. Chen H. Wu Y. Q. Li Y. H. (2023). Quality evaluation of Hawk tea from different months and regions based on quantitative analysis of multiple components with a single marker (QAMS) combined with HPLC fingerprint. Phytochem. Anal. 34 (7), 884–897. 10.1002/pca.3261 37483160

[B18] Mai J. Liang J. Liu X. Tan L. Xu H. Li Y. (2020). Simultaneous determination of 5 components in the leaves of Dimocarpus longan by quantitative analysis of multicomponents by single marker (QAMS) based on UPLC and HPLC. J. Anal. Methods. Chem. 2020 (1), 1–9. 10.1155/2020/3950609 PMC7204203 32399308

[B19] Men L. Liu Y. Qiu Y. Yuan X. (2021). An effective UPLC method for the quantification and fingerprint analysis of amides in a South China native medicinal herb, abri herba. J. Food. Compos. Anal. 96, 103723. 10.1016/j.jfca.2020.103723

[B20] Peng Y. Dong M. Zou J. Liu Z. (2018). Analysis of the HPLC fingerprint and QAMS for Sanhuang gypsum soup. J. Anal. Methods. Chem. 2018 (1), 1–14. 10.1155/2018/5890973 PMC6051103 30079260

[B21] Qu H. Ma Y. Yu K. Cheng Y. (2007). Simultaneous determination of eight active components in Chinese medicine ‘YIQING’capsule using high-performance liquid chromatography. J. Pharm. Biomed. Anal. 43 (1), 66–72. 10.1016/j.jpba.2006.06.013 16846714

[B22] Si W. Qiao Y. Liu Z. Jin G. Liu Y. Xue X. (2020). Combination of multi-model statistical analysis and quantitative fingerprinting in quality evaluation of shuang-huang-lian oral liquid. Anal. Bioanal. Chem. 412, 7073–7083. 10.1007/s00216-020-02841-z 32808053

[B23] Stekolshchikova E. Turova P. Shpigun O. Rodin I. Stavrianidi A. (2018). Application of quantitative analysis of multi-component system approach for determination of ginsenosides in different mass-spectrometric conditions. J. Chromatogr. A 1574 (82-90), 82–90. 10.1016/j.chroma.2018.09.005 30217383

[B24] Sun J. Tian F. Zhang Y. Wu M. Mao R. Le Z. (2019). Chromatographic fingerprint and quantitative analysis of commercial Pheretima aspergillum (Guang Dilong) and its adulterants by UPLC‐DAD. Int. J. Anal. Chem. 2019 (1), 1–10. 10.1155/2019/4531092 PMC6343145 30728838

[B25] Trawiński J. Skibiński R. (2019). Photodegradation study of sertindole by UHPLC-ESI-Q-TOF and influence of some metal oxide excipients on the degradation process. pharm 11 (7), 299. 10.3390/pharmaceutics11070299 PMC6680419 31252531

[B26] Wang C. Q. Jia X.-H. Zhu S. Komatsu K. Wang X. Cai S. Q. (2015). A systematic study on the influencing parameters and improvement of quantitative analysis of multi-component with single marker method using notoginseng as research subject. Talanta 134, 587–595. 10.1016/j.talanta.2014.11.028 25618711

[B27] Wang D. Gu X. Fang K. Fu B. Liu Y. Di X. (2023). Study on quality control of Zuojin pill by HPLC fingerprint with quantitative analysis of multi-components by single marker method and antioxidant activity analysis. J. Pharm. Biomed. Anal. 225, 115075. 10.1016/j.jpba.2022.115075 36603393

[B28] Wang J. Wang L. Lou G. H. Zeng H. R. Hu J. Huang Q. W. (2019). Coptidis Rhizoma: a comprehensive review of its traditional uses, botany, phytochemistry, pharmacology and toxicology. Pharm. Biol. 57 (1), 193–225. 10.1080/13880209.2019.1577466 30963783 PMC6461078

[B29] Wang Y. Yu Y. Sun G. Guo Y. (2020). Quality evaluation of powdered poppy capsule extractive by systematic quantified fingerprint method combined with quantitative analysis of multi-components by single marker method. J. Pharm. Biomed. Anal. 185, 113247. 10.1016/j.jpba.2020.113247 32193042

[B30] Wang Z. L. Wang S. Kuang Y. Hu Z. M. Qiao X. Ye M. (2018). A comprehensive review on phytochemistry, pharmacology, and flavonoid biosynthesis of Scutellaria baicalensis. Pharm. Biol. 56 (1), 465–484. 10.1080/13880209.2018.1492620 31070530 PMC6292351

[B31] Wu C. Xu B. Li Z. Song P. Chao Z. (2021). Gender discrimination of Populus tomentosa barks by HPLC fingerprint combined with multivariate statistics. Plant. Direct. 5 (3), e00311. 10.1002/pld3.311 33748656 PMC7963124

[B32] Xiong W. Yan R. Liu Y. Peng S. Jiang Z. Chai X. (2014). Establishment and validation of quantitative analysis of multi-components by single-marker for quality assessment of compound danshen preparations. Acta. Chromatogr. 26 (4), 695–710. 10.1556/achrom.26.2014.4.11

[B33] Xu X. Yang L. Zhao D. Wang Y. Dai L. Li S. (2024). New quality evaluation of Qizhi Xiangfu pills based on fingerprint with chemometric analysis and quantitative analysis of multi-components by single marker. J. Chromatogr. Sci. 62, 854–863. 10.1093/chromsci/bmae005 38446787

[B34] Yang L. Li Y. Hou Y. Wu Y. Tan L. Mu Z. (2023). Multicomponent analysis of Liuwei Dihuang pills by a single marker quantification method and chemometric discrimination of fingerprints. J. Anal. Methods. Chem. 2023, 1–14. 10.1155/2023/6648668 PMC10516694 37743973

[B35] Yang Y. Vong C. T. Zeng S. Gao C. Chen Z. Fu C. (2021). Tracking evidences of Coptis chinensis for the treatment of inflammatory bowel disease from pharmacological, pharmacokinetic to clinical studies. J. Ethnopharmacol. 268, 113573. 10.1016/j.jep.2020.113573 33181286

[B36] Zeng Y. Zhao L. Hao M. Maimaiti M. Li Z. Zhang M. (2024). Analysis of an aqueous extract from Turkish galls based on multicomponent qualitative and quantitative analysis combined with network pharmacology and chemometric analysis. J. Anal. Methods. Chem. 2024 (1), 1–18. 10.1155/2024/9962574 PMC11139529 38817340

[B37] Zhang C. Y. Li X. X. Li P. Jiang Y. Li H. J. (2021). Consistency evaluation between dispensing granule and traditional decoction from Coptidis Rhizoma by using an integrated quality‐based strategy. Phytochem. Anal. 32 (2), 153–164. 10.1002/pca.2905 31916640

[B38] Zhang F. Wu R. Liu Y. Dai S. Xue X. Li Y. (2023). Nephroprotective and nephrotoxic effects of Rhubarb and their molecular mechanisms. Biomed. Pharmacother. 160, 114297. 10.1016/j.biopha.2023.114297 36716659

[B39] Zhang R. T. Qing W. W. Yang L. Zou J. J. Shi Y. T. Xu X. L. (2022). Fingerprint combining with quantitative analysis of multi‐components by single marker for quality control of Chenxiang Huaqi tablets. Phytochem. Anal. 33 (3), 335–343. 10.1002/pca.3090 34693578

[B40] Zhang X. Wang L. Zong R. Zhang Z. Cheng F. Song C. (2024). A rapid protocol for distinguishing the quality of Sanshengyin and identifying potential markers by the “three-in-one” fingerprint profiles with antioxidant activity. J. Chromatogr. A 1740, 465553. 10.1016/j.chroma.2024.465553 39615417

[B41] Zheng G. D. Li K. Li Y. S. Liu E. H. (2012). Fast profiling of chemical constituents in Yiqing Capsule by ultra‐performance liquid chromatography coupled to electrospray ionization tandem mass spectrometry. J. Sep. Sci. 35 (1), 174–183. 10.1002/jssc.201100736 22125294

[B42] Zhu C. Li X. Zhang B. Lin Z. (2017). Quantitative analysis of multi-components by single marker—a rational method for the internal quality of Chinese herbal medicine. Integr. Med. Res. 6 (1), 1–11. 10.1016/j.imr.2017.01.008 28462138 PMC5395686

[B43] Zhu J. Zhu F. Li L. Cheng L. Zhang L. Sun Y. (2019). Highly discriminant rate of Dianhong black tea grades based on fluorescent probes combined with chemometric methods. Food. Chem. 298, 125046. 10.1016/j.foodchem.2019.125046 31260981

[B44] Zou J. J. Xu X. L. Yang L. Wang Y. W. Li Y. Dai L. (2023). Comprehensive quality evaluation of qizhi xiangfu pills based on quantitative analysis of multi-components by a single marker combined with GC fingerprints and chemometrics. J. AOAC. Int. 106 (5), 1414–1423. 10.1093/jaoacint/qsad043 37027226

