# Supplementary material for: UPLC fingerprinting combined with quantitative analysis of multicomponents by a single marker for quality evaluation of YiQing granules
Source: Front Chem. 2025 Jul 28;13:1632033. doi: 10.3389/fchem.2025.1632033 (PMC12336232; doi:10.3389/fchem.2025.1632033)
Supplement: Supplementary file 1 [file Table1.docx]

| **Table S1 Comparison of the contents (mg/g) of twelve components in YQGS determined by quantitative analysis of QAMS and ESM** | | | | | | | | | | | | | |
| --- | --- | --- | --- | --- | --- | --- | --- | --- | --- | --- | --- | --- | --- |
| Sample | Berberine | Epiberberine | | | Coptisine | | | Palmatine | | | Baicalin | | |
|  | ESM | QAMS | ESM | SMD% | QAMS | ESM | SMD% | QAMS | ESM | SMD% | QAMS | ESM | SMD% |
| 1 | 1. 441 | 0. 412 | 0. 420 | 1.961 | 0. 271 | 0. 272 | -0.289 | 0. 350 | 0. 351 | -0.093 | 5. 152 | 5. 049 | 2.005 |
| 2 | 1. 702 | 0. 437 | 0. 446 | 1.936 | 0. 284 | 0. 285 | -0.377 | 0. 402 | 0. 403 | -0.104 | 2. 882 | 2. 804 | 2.697 |
| 3 | 0. 298 | 0. 130 | 0. 136 | 4.558 | 0. 036 | 0. 038 | -4.712 | 0. 071 | 0. 072 | -0.518 | 4. 970 | 4. 727 | 4.872 |
| 4 | 1. 050 | 0. 302 | 0. 310 | 2.651 | 0. 206 | 0. 206 | -0.330 | 0. 258 | 0. 258 | -0.114 | 2. 086 | 2. 004 | 3.916 |
| 5 | 1. 630 | 0. 417 | 0. 426 | 2.028 | 0. 276 | 0. 277 | -0.376 | 0. 416 | 0. 416 | -0.040 | 4. 034 | 3. 946 | 2.190 |
| 6 | 1. 320 | 0. 430 | 0. 437 | 1.761 | 0. 268 | 0. 268 | -0.219 | 0. 316 | 0. 316 | -0.120 | 1. 982 | 1. 907 | 3.790 |
| 7 | 1. 149 | 0. 395 | 0. 402 | 1.854 | 0. 270 | 0. 270 | -0.056 | 0. 280 | 0. 280 | -0.116 | 4. 971 | 4. 858 | 2.282 |
| 8 | 0. 471 | 0. 156 | 0. 163 | 4.675 | 0. 084 | 0. 085 | -1.071 | 0. 121 | 0. 121 | -0.113 | 2. 782 | 2. 647 | 4.850 |
| 9 | 0. 326 | 0. 133 | 0. 140 | 4.710 | 0. 054 | 0. 055 | -2.005 | 0. 082 | 0. 082 | -0.263 | 6. 729 | 6. 394 | 4.985 |
| 10 | 1. 249 | 0. 388 | 0. 396 | 1.890 | 0. 253 | 0. 253 | -0.126 | 0. 321 | 0. 320 | 0.062 | 7. 387 | 7. 244 | 1.935 |
| 11 | 0. 558 | 0. 201 | 0. 208 | 3.467 | 0. 097 | 0. 098 | -0.998 | 0. 133 | 0. 134 | -0.286 | 3. 535 | 3. 394 | 3.982 |
| 12 | 0. 727 | 0. 199 | 0. 207 | 4.066 | 0. 134 | 0. 135 | -0.614 | 0. 184 | 0. 184 | -0.108 | 3. 004 | 2. 893 | 3.690 |
| 13 | 0. 992 | 0. 274 | 0. 282 | 2.968 | 0. 188 | 0. 189 | -0.409 | 0. 249 | 0. 249 | -0.088 | 3. 584 | 3. 480 | 2.895 |
| 14 | 1. 391 | 0. 397 | 0. 405 | 2.036 | 0. 259 | 0. 260 | -0.311 | 0. 334 | 0. 334 | -0.110 | 4. 943 | 4. 840 | 2.084 |
| 15 | 0. 859 | 0. 238 | 0. 247 | 3.390 | 0. 155 | 0. 156 | -0.577 | 0. 212 | 0. 212 | -0.133 | 2. 341 | 2. 249 | 3.919 |
| 16 | 0. 398 | 0. 140 | 0. 147 | 4.987 | 0. 049 | 0. 051 | -3.372 | 0. 080 | 0. 081 | -1.094 | 1. 844 | 1. 906 | -3.379 |
| 17 | 0. 117 | 0. 178 | 0. 186 | 4.581 | 0. 075 | 0. 077 | -2.415 | 0. 054 | 0. 057 | -4.675 | 2. 252 | 2. 152 | 4.453 |

| Sample | Wogonoside | | | Baicalein | | | Wogonin | | | Aloe-emodin | | |
| --- | --- | --- | --- | --- | --- | --- | --- | --- | --- | --- | --- | --- |
|  | QAMS | ESM | SMD% | QAMS | ESM | SMD% | QAMS | ESM | SMD% | QAMS | ESM | SMD% |
| 1 | 0. 859 | 0. 838 | -2.432 | 0. 291 | 0. 292 | 0.218 | 0. 142 | 0. 140 | -1.456 | 0. 010 | 0. 010 | -1.893 |
| 2 | 0. 352 | 0. 336 | -4.489 | 0. 066 | 0. 069 | 4.126 | 0. 022 | 0. 021 | -4.157 | 0. 005 | 0. 005 | -2.899 |
| 3 | 1. 256 | 1. 198 | -4.799 | 0. 243 | 0. 235 | -3.267 | 0. 147 | 0. 142 | -3.077 | 0. 022 | 0. 022 | -2.353 |
| 4 | 0. 421 | 0. 404 | -4.396 | 0. 055 | 0. 058 | 4.634 | 0. 027 | 0. 026 | -4.023 | 0. 003 | 0. 003 | -4.360 |
| 5 | 1. 059 | 1. 038 | -2.034 | 0. 413 | 0. 413 | -0.013 | 0. 128 | 0. 126 | -1.398 | 0. 053 | 0. 053 | -1.022 |
| 6 | 0. 428 | 0. 411 | -4.064 | 0. 123 | 0. 125 | 1.689 | 0. 048 | 0. 047 | -2.545 | 0. 005 | 0. 005 | -3.134 |
| 7 | 1. 100 | 1. 074 | -2.354 | 0. 127 | 0. 129 | 1.463 | 0. 054 | 0. 053 | -2.523 | 0. 019 | 0. 019 | -1.700 |
| 8 | 0. 725 | 0. 691 | -4.937 | 0. 102 | 0. 103 | 0.418 | 0. 038 | 0. 036 | -4.960 | 0. 007 | 0. 007 | -4.346 |
| 9 | 0. 513 | 0. 489 | -4.929 | 0. 223 | 0. 217 | -2.931 | 0. 117 | 0. 111 | -4.978 | 0. 010 | 0. 009 | -4.645 |
| 10 | 1. 367 | 1. 339 | -2.138 | 0. 223 | 0. 224 | 0.428 | 0. 066 | 0. 064 | -2.307 | 0. 031 | 0. 031 | -1.522 |
| 11 | 0. 687 | 0. 658 | -4.411 | 0. 176 | 0. 175 | -0.517 | 0. 096 | 0. 093 | -3.267 | 0. 009 | 0. 008 | -3.654 |
| 12 | 0. 578 | 0. 555 | -4.163 | 0. 055 | 0. 057 | 4.155 | 0. 025 | 0. 024 | -4.851 | 0. 011 | 0. 011 | -2.795 |
| 13 | 0. 797 | 0. 773 | -2.999 | 0. 269 | 0. 269 | -0.111 | 0. 121 | 0. 119 | -1.981 | 0. 004 | 0. 003 | -4.186 |
| 14 | 0. 844 | 0. 823 | -2.494 | 0. 300 | 0. 300 | 0.151 | 0. 138 | 0. 136 | -1.503 | 0. 008 | 0. 008 | -2.172 |
| 15 | 0. 435 | 0. 416 | -4.611 | 0. 102 | 0. 104 | 1.701 | 0. 041 | 0. 040 | -3.393 | 0. 004 | 0. 004 | -3.949 |
| 16 | 0. 923 | 0. 879 | -4.981 | 0. 078 | 0. 079 | 0.892 | 0. 055 | 0. 052 | -4.916 | 0. 007 | 0. 007 | -4.954 |
| 17 | 0. 584 | 0. 559 | -4.308 | 0. 086 | 0. 088 | 1.869 | 0. 041 | 0. 039 | -3.868 | 0. 015 | 0. 014 | -2.736 |

| Sample | Rhein | | | Emodin | | | Chrysophanol | | |
| --- | --- | --- | --- | --- | --- | --- | --- | --- | --- |
|  | QAMS | ESM | SMD% | QAMS | ESM | SMD% | QAMS | ESM | SMD% |
| 1 | 0. 062 | 0. 065 | 4.911 | 0. 010 | 0. 009 | -1.639 | 0. 015 | 0. 015 | -4.936 |
| 2 | 0. 062 | 0. 065 | 4.984 | 0. 009 | 0. 009 | -1.527 | 0. 016 | 0. 015 | -4.669 |
| 3 | 0. 146 | 0. 143 | -2.004 | 0. 028 | 0. 027 | -3.856 | 0. 022 | 0. 021 | -4.647 |
| 4 | 0. 051 | 0. 054 | 4.686 | 0. 002 | 0. 002 | -4.323 | 0. 009 | 0. 009 | -3.078 |
| 5 | 0. 107 | 0. 110 | 2.621 | 0. 023 | 0. 023 | -1.118 | 0. 053 | 0. 052 | -1.963 |
| 6 | 0. 085 | 0. 088 | 3.162 | 0. 003 | 0. 003 | -3.198 | 0. 034 | 0. 033 | -2.952 |
| 7 | 0. 072 | 0. 075 | 3.921 | 0. 012 | 0. 012 | -1.722 | 0. 031 | 0. 030 | -3.151 |
| 8 | 0. 053 | 0. 055 | 4.060 | 0. 008 | 0. 007 | -3.859 | 0. 017 | 0. 016 | -4.999 |
| 9 | 0. 045 | 0. 047 | 4.062 | 0. 014 | 0. 014 | -4.841 | 0. 044 | 0. 043 | -2.785 |
| 10 | 0. 386 | 0. 385 | -0.218 | 0. 038 | 0. 037 | -1.381 | 0. 032 | 0. 031 | -3.101 |
| 11 | 0. 067 | 0. 069 | 3.131 | 0. 005 | 0. 005 | -3.854 | 0. 025 | 0. 024 | -4.990 |
| 12 | 0. 089 | 0. 091 | 2.301 | 0. 012 | 0. 012 | -2.465 | 0. 021 | 0. 020 | -4.890 |
| 13 | 0. 059 | 0. 062 | 4.788 | 0. 004 | 0. 004 | -3.182 | 0. 017 | 0. 016 | -4.996 |
| 14 | 0. 066 | 0. 069 | 4.510 | 0. 009 | 0. 009 | -1.741 | 0. 017 | 0. 016 | -4.571 |
| 15 | 0. 066 | 0. 069 | 3.946 | 0. 005 | 0. 005 | -2.825 | 0. 021 | 0. 020 | -4.560 |
| 16 | 0. 054 | 0. 056 | 3.442 | 0. 005 | 0. 005 | -4.878 | 0. 043 | 0. 041 | -5.002 |
| 17 | 0. 054 | 0. 056 | 4.784 | 0. 013 | 0. 013 | -2.576 | 0. 043 | 0. 042 | -3.471 |
| Note: SMD (%)=$\frac{ESM-QAMS}{\mathrm{ESM}}$*100% | | | | | | | | | |
